# A LAPS-Based Differential Sensor for Parallelized Metabolism Monitoring of Various Bacteria

**DOI:** 10.3390/s19214692

**Published:** 2019-10-29

**Authors:** Shahriar Dantism, Désirée Röhlen, Torsten Wagner, Patrick Wagner, Michael J. Schöning

**Affiliations:** 1 Institute of Nano- and Biotechnologies (INB), FH Aachen, Heinrich-Mußmann-Straße 1, 52428 Jülich, Germany; dantism@fh-aachen.de (S.D.); roehlen@fh-aachen.de (D.R.); torsten.wagner@fh-aachen.de (T.W.); 2 Department of Physics and Astronomy, Laboratory for Soft Matter and Biophysics, KU Leuven, Celestijnenlaan 200 D, 3001 Leuven, Belgium; patrickhermann.wagner@kuleuven.be; 3 Institute of Complex Systems (ICS-8), Research Centre Jülich GmbH, Wilhelm-Johnen-Straße 1, 52425 Jülich, Germany

**Keywords:** light-addressable potentiometric sensor (LAPS), *Lactobacillus brevis*, *Escherichia coli*, *Corynebacterium glutamicum*, cellular metabolism, differential cell-based measurement, multi-analyte analysis, extracellular acidification

## Abstract

Monitoring the cellular metabolism of bacteria in (bio)fermentation processes is crucial to control and steer them, and to prevent undesired disturbances linked to metabolically inactive microorganisms. In this context, cell-based biosensors can play an important role to improve the quality and increase the yield of such processes. This work describes the simultaneous analysis of the metabolic behavior of three different types of bacteria by means of a differential light-addressable potentiometric sensor (LAPS) set-up. The study includes *Lactobacillus brevis*, *Corynebacterium glutamicum*, and *Escherichia coli*, which are often applied in fermentation processes in bioreactors. Differential measurements were carried out to compensate undesirable influences such as sensor signal drift, and pH value variation during the measurements. Furthermore, calibration curves of the cellular metabolism were established as a function of the glucose concentration or cell number variation with all three model microorganisms. In this context, simultaneous (bio)sensing with the multi-organism LAPS-based set-up can open new possibilities for a cost-effective, rapid detection of the extracellular acidification of bacteria on a single sensor chip. It can be applied to evaluate the metabolic response of bacteria populations in a (bio)fermentation process, for instance, in the biogas fermentation process.

## 1. Introduction

Food digestion within the gastrointestinal tract is a good example for biofermentation processes in our daily life, in which many different types of microorganisms can be involved. Without microbes, large and complex food molecules cannot be broken down into required nutrients for the human body [1]. In the food-research sector, complex microbial interactions have been studied to enhance, for instance, the aroma profile and flavor in soy sauce during the fermentation process [2]. As another example, microorganisms play a major role in coffee fermentation by degrading the mucilage to alcohols, acids, and enzymes [3]. In agricultural biogas plants, various types of bacteria contribute to the conversion of biomass (e.g., maize silage) into a usable energy source (e.g., methane gas) [4]. In all applications of bioreactor technology, the on-line monitoring of the metabolic activity of microorganisms should be seriously considered to avoid undesired, time-consuming, and cost-intensive interventions, which can reduce the yield at the end of the production chain. Related fields of application include: cell health monitoring in bioreactors [5], continuous non-invasive monitoring of cell growth in disposable bioreactors [6], on-line near-infrared bioreactor monitoring of cell density [7], online monitoring of cell concentration in high-cell density *Escherichia coli* cultivations [8], sensing metabolites for monitoring the tissue-engineered cellularity in perfusion bioreactors [9], and micro-biosensors for fed-batch fermentation with integrated online monitoring [10]. In all of those examples, analytical sensors are the enabling element for rapid, sensitive, and cost-effective detection of various parameters (e.g., health, growth, and density of cells in bioreactors). In this context, light-addressable potentiometric sensors (LAPS) can be applied as suitable tools for monitoring the extracellular acidification of cells. A LAPS is a field-effect-based chemical sensor with the ability to monitor concentration changes of biochemical/biological species in a spatially resolved way [11,12,13]. It belongs to the family of electrolyte/insulator/semiconductor (EIS)-based capacitive sensors [14]. By addressing defined regions of interest on a sensor chip with modulated light beams such as laser-diode modules, two-dimensional (2D) chemical images of concentration distributions can be recorded [15,16,17,18]. In comparison to other 2D potentiometric chemical imaging sensors applying e.g., arrays of ion-sensitive field-effect transistors (ISFETs) [19] or charge-coupled devices (CCDs) [20], LAPS require no sensor patterns to record chemical images: The LAPS surface does not require pattering, wiring or passivation, which allows bacteria to come directly into contact with the pH-sensitive transducer layer to determine the extracellular acidification. Furthermore, on its planar sensor surface, multi-chamber structures can be attached to perform differential measurements with distinct cell suspensions [21]. The principle of differential measurements allows elimination of unwanted external influences such as sensor signal drift and pH value variations of the measurement solution during experiments [22]. Beside the chemical imaging technique visualizing the pH distribution on the sensor surface, there are a variety of further LAPS sensing techniques such as the scanned light-pulse technique (SLPT) in studies of interface properties (e.g*.,* flat-band voltage) [15], or scanning photo-induced impedance microscopy (SPIM) analyzing impedance changes e.g*.,* in PAH/PSS polyelectrolyte microcapsules labeled with Au nanoparticles [23]. The equivalent circuit diagram for designing the LAPS set-up consists of three key parts (illumination area, non-illumination area, and an external circuit), which are described in details in [15]. The sensor fabrication steps are described in Section 2.1. Explanations about LAPS operation modes (e.g., constant-bias, constant-current, potential-tracking, and phase-mode) can be found in [24].

A few examples of on-going research for LAPS-based biosensing are the determination of the extracellular acidification of *Escherichia coli* [25,26,27], and differential imaging of the metabolism of bacteria and eukaryotic cells [28]. In this regard, quantitative differential monitoring of the metabolic activity of *Corynebacterium glutamicum* [29], and image detection of yeast *Saccharomyces cerevisiae* [30] have been recently discussed. Correspondingly, dual functional extracellular recording for better signal transduction [31], and monitoring secretion of adrenal chromaffin cells by local extracellular acidification [32] are further applications.

In this work and for the first time, the extracellular acidification of three model microorganisms, namely *Escherichia coli* (*E. coli*) K12, *Corynebacterium glutamicum* (*C. glutamicum*) ATCC13032, and *Lactobacillus brevis* (*L. brevis*) ATCC 14869 is assessed simultaneously by means of a four-chamber differential LAPS set-up by varying the cell number and/or glucose concentration. These model microorganisms were selected, as acid-forming, facultative anaerobe, easy-to-cultivate, and commonly used bacteria in laboratory and industrial applications [25,33,34]. Both *E. coli* and *C. glutamicum* have been studied in separate LAPS experiments recently [27,29], however, the cellular metabolism of *L. brevis* has not been analyzed by LAPS so far. In a first experimental step, the extracellular acidification of *L. brevis* cells has been evaluated: calibration curves were established, which render the potential-change rate as a function of glucose concentration and cell number. Data were compared with results of already in literature discussed microorganisms such as *E. coli* and *C. glutamicum*. Finally, a novel parallelized measurement procedure is introduced, which allows sequentially and simultaneously performed LAPS measurements with all three types of bacteria.

The motivation is briefly explained as follows: In terms of the specific metabolic characteristic of each model microorganism, distinguishable sensor signal responses with LAPS can be obtained. This way, different signal patterns of studied bacteria can be saved in a database. Later on, such data can be applied as references to evaluate the cellular metabolism of bacteria populations in a fermentation process (e.g., in biogas processes). Here, the mutual metabolic influence of cells in the fermenter broth on the metabolization of model microorganisms can be studied. Hence, signal variations after ‘interactions’ between bacteria can be detected. This approach can contribute to a better understanding of the fermentation process and help to avoid bacteria-related process crashes in a bioreactor. In addition, the multi-analyte differential measurement on a single LAPS chip underlines the possibility of combinatorial analysis with different cell types in parallel enabling a fast data collection utilizing a capacitive field-effect biosensor.

## 2. Materials and Methods

### 2.1. Sensor Fabrication and Measurement Set-Up

**Figure 1:**
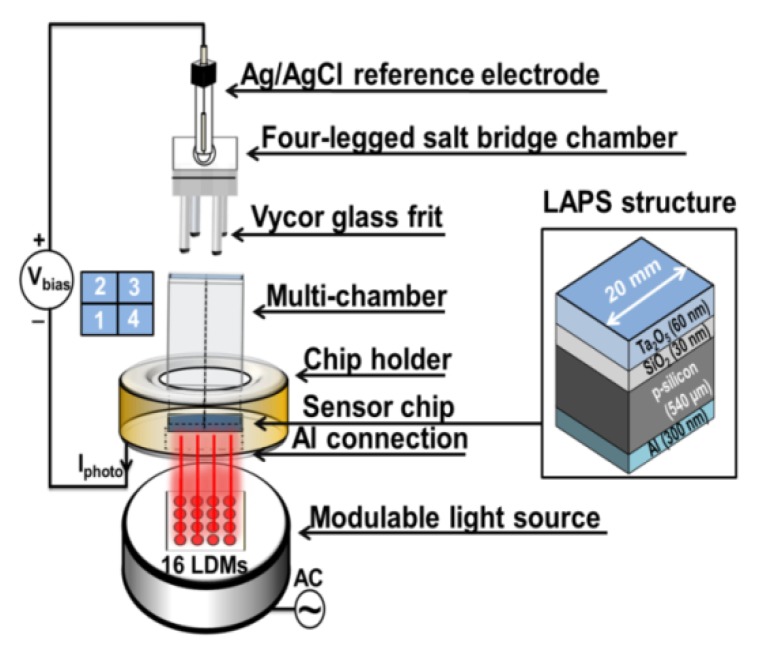
Schematic illustration of the four-chamber differential LAPS measurement set-up, consisting of an Ag/AgCl reference electrode, a PP-ABS four-legged salt bridge chamber (total height 40 mm, leg height 20 mm, Ø_legs_ 4 mm, container area = 15 × 15 mm^2^) combined with four Vycor glass frits (Ø_frit_ 3.2 mm, height 4 mm) filled with 1 mL KCl solution (3 M), a PP-ABS-based four-chamber structure (sensing area per chamber ≈ 7 × 7 mm^2^, height 20 mm), a chip holder (Ø 40 mm, height 10 mm) made of PEEK, a LAPS chip with Al/p-Si/SiO_2_/Ta_2_O_5_ layers, and a light source based on an array containing 16 infrared laser-diode modules (4 per chamber, Ø 3.3 mm, length 7 mm). Chambers 1, 2, and 3 are used as active sensor side with cells. Chamber 4 serves as a reference chamber without cells. The active rear-side illumination area is 15 × 15 mm^2^. I_photo_: photocurrent, V_bias_: bias voltage, LDMs: laser-diode modules, AC: alternating current.

The LAPS chip consists of an Al/p-Si/SiO_2_/Ta_2_O_5_ field-effect structure: Starting with a p-doped silicon wafer (<100>, 5–10 Ωcm, thickness: 540 µm), 30 nm SiO_2_ were grown by a thermal dry oxidation step (O_2_, 40 min at 1000 °C), followed by deposition of the Ta_2_O_5_ layer (electron-beam evaporation (0.5 nm/s, at 6 × 10^−6^ mbar) of 30 nm Ta and subsequent oxidation (45 min at 520 °C) to 60 nm Ta_2_O_5_). The ohmic rear-side contact of 300 nm Al on Si was also prepared by electron-beam evaporation. Subsequently, the silicon wafer was diced into single LAPS chips of 20 × 20 mm^2^ size, and a part of the rear-side contact was removed by wet-chemical etching (5% hydrofluoric acid) defining a window for the rear-side illumination. The sensor fabrication steps are described in detail in the references [35,36,37,38]. The LAPS chip was mounted in a home-made measurement cell and the set-up is schematically illustrated in Figure 1.

The measurement cell consists of a 3D-printed photopolymer-based (polypropylene-acrylonitrile-butadiene-styrene, PP-ABS) four-chamber structure combined with a four-legged salt bridge chamber (filled with 1 mL of 3 M KCl solution) and a polymer-based (polyether ether ketone, PEEK) chip holder, housing the LAPS chip as working electrode and a commercial Ag/AgCl (Metrohm GmbH) reference electrode. More information related to the design, size, and performance of all constructed polymer structures can be found in previous publications [19,27,28]. The four-chamber structure enables to study different cell types simultaneously. In the following experiment, three chambers (1–3) serve as active sensor site with various cell suspensions, while the fourth chamber was used as a reference chamber without cells. This way, pH- and temperature fluctuations as well as a signal drift of the sensor can be compensated. To read out the LAPS sensor signal, a DC (direct current) bias voltage (V_bias_) is applied between the Ag/AgCl reference electrode and the rear-side contact. The illumination unit is based on an array of 16 small-sized, fixed-focus, and tunable infrared laser-diode modules (LDMs, λ = 785 nm, Roithner Lasertechnik GmbH, Vienna, Austria, serial number APCD-780-07-C3). A simultaneous modulation with different frequencies (frequency divider with a constant value of 160, main clock: 160 MHz, sampling frequency: 1 MHz) was carried out through a field-programmable gate array (FPGA)-based microcontroller, see details in [39]; the illuminated and non-illuminated sensor areas can be modeled with equivalent circuit diagrams as discussed in [40].

The bias voltage V_bias_ allows to induce the formation of a space-charge region within the p-doped silicon at the insulator/semiconductor interface [41]. Here, the charge carriers, i.e., electron-hole pairs induced by the light source, are separated in the electrical field, resulting in a photocurrent, I_photo_. This photocurrent depends on the surface potential of the LAPS chip. Due to the direct contact of the pH-sensitive Ta_2_O_5_ transducer layer with the microorganisms in the analyte (chambers 1, 2, and 3), changes in their metabolic activity (extracellular acidification) will consequently lead to changes in the surface potential through a variation of the H^+^-ion activity of the LAPS surface [21]. Besides of current–voltage (I–V) measurements, the LAPS chips were electrochemically characterized by capacitance–voltage- (C–V), impedance spectroscopy-, leakage-current-, and constant-capacitance (ConCap) measurements utilizing an electrochemical spectrum analyzer (Zahner-Elektrik GmbH). The sensitivity of the transducer structure (54 mV/pH) was determined as described in [27]. Further information about the electrochemical characterization of the LAPS chips and the FPGA-based set-up can be also found in [42,43,44,45].

### 2.2. Sample Preparation and Microorganism Cultivation

For the preparation of glucose solutions and cell suspensions, diluted phosphate-buffered saline (PBS) was used as stock solution: Here, 0.2 g of KCl, 8 g of NaCl, 1.15 g of Na_2_HPO_4_, and 0.2 g of KH_2_PO_4_ were dissolved in 1 L distilled water. The buffer solution was further diluted with distilled water, so that a total buffer capacity of 0.2 mM was obtained. A low-buffer capacity is required to observe the extracellular acidification of cells on the LAPS. To determine the buffer capacity, a titration method was used with hydrochloric acid (HCl, 1 M). The buffer was autoclaved at 121 °C for 2 h. Subsequently, the pH value was adjusted at pH 7.4 using NaOH/HCl (1 M) solution. Different glucose concentrations (1.67, 2.5, 3.33, and 5 mM) were prepared after a serial dilution of the stock glucose solution (10 mM) utilizing the diluted PBS. The procedure to cultivate Gram-negative, rod-shaped *E. coli* K12 bacteria (24 × 10^9^ CFU/mL cells) is described in [21,27]. Gram-positive, rod-shaped *C. glutamicum* ATCC13032 bacteria were cultivated on a *Corynebacterium* agar consisting of 10 g of casein peptone, 5 g of yeast extract, 5 g of glucose, 5 g of NaCl, 15 g of agar, in 1 L distilled water. First, cells were incubated at 30 °C by 141 rpm for about 6 h. Optical density measurements (Fisher Scientific, GE Healthcare Ultraspec^TM^ 2100 pro) were used to determine the cell growth density (λ = 578 nm) in all cell suspensions. The overnight cultivation was performed in an incubator around 12 h at 30 °C. This way, 24 × 10^9^ CFU/mL cells were harvested after two washing steps with the diluted PBS solution. After the centrifugation step, the cell pellet was resuspended in the buffer solution (pH 7.4, 0.2 mM).

For the cultivation of Gram-positive, heterofermentive, rod-shaped *L. brevis* ATCC 14869 bacteria, MRS- (De Man, Rogosa and Sharpe) selective culture medium was applied. The MRS agar contains 10 g of casein, 10 g of meat extract, 5 g of yeast extract, 20 g of glucose, 1 g of TWEEN 80, 2 g of K_2_HPO_4_, 5 g of Na-acetate, 0.2 g MgSO_4_ × 7 H_2_O, 0.05 g of MNSO_4_ × H_2_O, and 1 L of distilled water. The pH value was adjusted to pH 6.2. Cells were incubated at 30 °C by for about 48 h. Further steps are similar to the cultivation of *C. glutamicum* cells, as mentioned above to obtain 24 × 10^9^ CFU/mL cells.

With all cultivated microorganisms, five different cell numbers (0.3 × 10^9^, 0.6 × 10^9^, 1.2 × 10^9^, 2.4 × 10^9^, 4.8 × 10^9^ cells in 200 µL suspension) were applied for the four-chamber differential LAPS measurements by a 1:2 dilution series with PBS solution to study their metabolic behavior. The as-prepared cell suspensions were used on the same day, in which LAPS measurements were performed. Freshly cultured suspensions were necessary for each measurement day to guarantee the reproducibility of the experiments. All final pH values of the cell suspensions were adjusted to 7.4 before starting with the cell-based differential LAPS measurements and controlled by a conventional pH-glass electrode (type: DGi115-SC, Mettler Toledo, Zurich, Switzerland).

## 3. Results and Discussion

### 3.1. Monitoring the Cellular Metabolism of L. Brevis Bacteria

**Figure 2:**
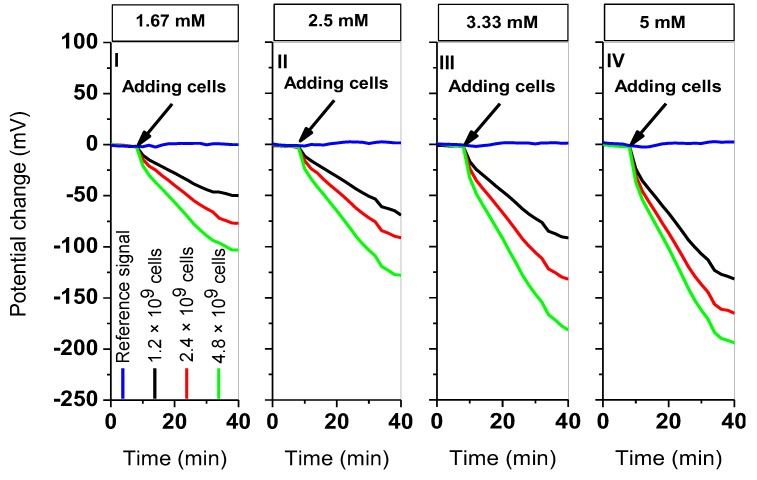
Four-chamber differential LAPS measurement with varying cell number of *L. brevis* (1.2 × 10^9^, 2.4 × 10^9^, 4.8 × 10^9^ cells in 200 µL cell suspension) and varying glucose concentration (1.67, 2.5, 3.33, 5 mM). Potential changes of four successive independent measurements with an ascending series of glucose concentrations. Blue line: reference sensor signal without cells; black, red, and green lines: active sensor sites with cells. Four laser-diode modules (LDMs) were considered for each chamber (each curve corresponds to calculated mean values).

The analysis of the metabolic activity of *E. coli* and *C. glutamicum* has been recently reported in [27,29]. This section describes the determination of cellular metabolism of *L. brevis* cell suspensions utilizing the four-chamber differential LAPS set-up. Three different cell numbers (1.2 × 10^9^, 2.4 × 10^9^, 4.8 × 10^9^ cells) were chosen to monitor the acidification behavior. In the first measurement (Figure 2, at 1.67 mM), all chambers were loaded with 100 µL glucose (end resulting concentration: 1.67 mM, pH 7.4). The sensor chip was first conditioned with the glucose solution for 10 min, then 200 µL of cell suspension (pH 7.4) were added into chambers 1, 2, and 3 and 200 µL of the diluted PBS solution in the reference chamber 4 (see set-up in Figure 1). Thus, a total suspension volume per chamber of 300 µL was used. For each chamber, four light spots in the area of interest were selected to read-out the sensor signal. After that, the mean values of the recorded potential change values per chamber were plotted (see diagram I). This measurement procedure was repeated for three further glucose concentrations (2.5, 3.33, and 5 mM) shown in the diagrams II, III, and IV.

**Figure 3:**
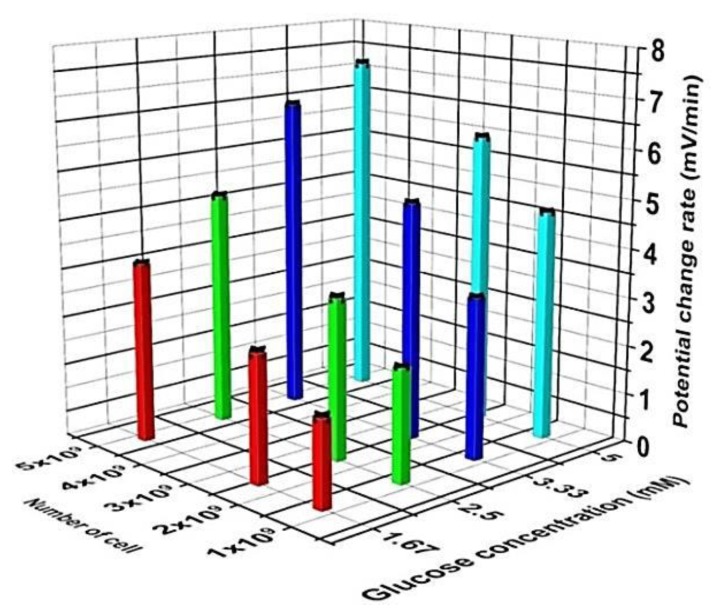
3D plot of mean values and standard deviations (three repetitions) of the potential change rates for four successive independent measurements with different glucose concentrations (1.67, 2.5, 3.33, 5 mM) when varying the cell numbers (1.2 × 10^9^, 2.4 × 10^9^, 4.8 × 10^9^ cells) in 200 µL of *L. brevis* cell suspension. Data are calculated from Figure 2. Mean values and standard deviations of PCR values were obtained from three independent measurement repetitions within the first 6 min after adding cells. For each chamber, four laser-diode modules (LDMs) were used. The mean value of signals per chamber was considered for further evaluations.

Figure 2 depicts the mean values of the potential changes *vs.* time in four independent successive measurements performed with increasing both the glucose concentration and the cell number. After each independent measurement of 40 min (first, the sensor chip was conditioned for 10 min without cells; then, the respective cell concentration was added), the sensor chip was washed with the diluted PBS solution (pH 7.4, 0.2 mM) and the next glucose concentration (e.g., 2.5 mM) was pipetted into the sensor chambers. Four output signals from four chambers are marked in different colors: in the first measurement at 1.67 mM glucose (diagram I), the blue line indicates the reference signal in the absence of cells in chamber 4. The black line refers to the cell suspension with the lowest cell number of 1.2 × 10^9^ cells, which results in a potential drop of about 50 mV. The signal from the chamber with 2.4 × 10^9^ cells is shown in red and indicates a potential change of approximately 77 mV. The highest signal change of approximately 103 mV with 4.8 × 10^9^ cells in chamber 3 is plotted in green, which corresponds to a pH value of ca. 5.5 on the sensor surface when considering a pH sensitivity of 54 mV/pH of the LAPS chip without cell suspensions [27]. In the measurement with the highest glucose concentration of 5 mM, the highest potential drop of approximately 194 mV was observed again in chamber 3 (green line) with 4.8 × 10^9^ cells, which corresponds to a pH shift at the LAPS surface of ΔpH ≈ 3.8. Further potential change values of 131 mV and 164 mV were achieved for cell numbers 1.2 × 10^9^ and 2.4 × 10^9^ cells in chamber 1 and chamber 2, respectively. For all measurements, the metabolic response of the acid-forming *L. brevis* bacteria induces potential changes after adding cells, which leads to an increase of H^+^-ion activity on the transducer surface. The extracellular acidification due to the metabolic activity causes a shift to the negative V_bias_ axis and a respective potential drop. The experiments have shown that by increasing the cell number and/or the glucose concentration in each measurement, higher potential change values can be achieved, as a result of metabolically active microorganisms and comparable to results obtained with *E. coli* and *C. glutamicum*, respectively [27,29]. From Figure 2, differential signals can be evaluated by subtracting the signal values of chambers with cells from the reference chamber without cells. The slope of the decreasing differential signals can be calculated in a particular time period (here, within the first 6 min after adding cells, i.e., from minute 10 to 16). These values describe the potential change rates (PCR) given in mV/min and can be computed through a linear regression method. Figure 3 represents the corresponding 3D plot of the calculated PCR values for different cell numbers (1.2 × 10^9^, 2.4 × 10^9^, and 4.8 × 10^9^ cells) and glucose concentrations (1.67, 2.5, 3.33, and 5 mM). For the detailed values, see Table S1 in supplementary information of this article.

By increasing the cell number and/or glucose concentration, the extracellular acidification and the PCR values increase. For the lowest cell number of 1.2 × 10^9^ cells at 1.67 mM glucose, the lowest PCR value of 1.76 ± 0.05 mV/min was calculated. At the highest applied glucose concentration of 5 mM and the highest cell number of 4.8 × 10^9^ cells, the highest LAPS signal response of 7.20 ± 0.05 mV/min was recorded.

### 3.2. Determination of Calibration Curves for L. brevis, C. Glutamicum, and E. Coli

**Figure 4:**
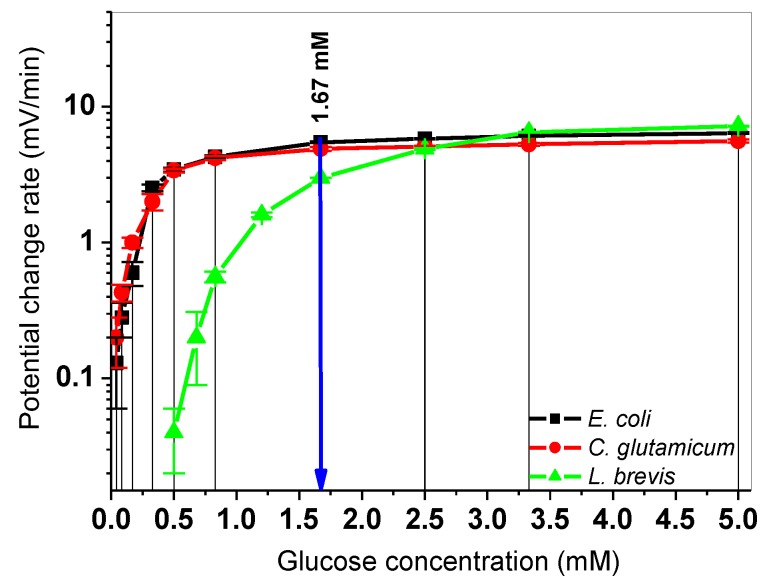
Correlations between the LAPS signal response and the glucose concentration. Mean values and standard deviations of PCR values of *E. coli* (black), *C. glutamicum* (red), and *L. brevis* (green) are depicted for different glucose concentrations (0.042, 0.085, 0.17, 0.20, 0.33, 0.40, 0.50, 0.68, 0.83, 1.20, 1.67, 2.50, 3.33, and 5 mM) at a constant cell number of 4.8 × 10^9^ cells. Three independent measurements were performed within the first 6 min after adding cells to calculate the mean PCR values. The blue arrow in the diagram indicates a particular glucose concentration (1.67 mM), which was exemplarily chosen to compare calibration curves of metabolic responses of the three microorganisms by cell number variations, see also Figure 5.

In this section, the PCR values of three microorganisms, namely *L. brevis*, *C. glutamicum*, and *E. coli*, are compared with each other by means of calibration curves as a function of glucose concentration or cell number. Different types of bacteria induce different independent LAPS signal responses due to their diverse acidification behavior on the sensor surface. With the knowledge of their signal characteristics (calibration curves), these model microorganisms might be used to analyze metabolic responses of microorganism populations within a (bio)fermentation broth (e.g., from a biogas reactor). It can be studied, whether/how cells in the fermentation broth from different process stages will influence the extracellular acidification of studied model bacteria: calibration matrices can be defined that later-on can be correlated with different scenarios of the biogas process. In this way, an external feedback control of the biogas operation might be envisaged. In our experiments, two approaches were separately considered: First, glucose concentrations were varied at a constant cell number (4.8 × 10^9^ cells) to find a correlation between the sensor signal and the glucose uptake. Second, calibration curves were defined by varying the particular cell number at a constant glucose concentration (1.67 mM). For the determination of the PCR values of the cellular metabolism of *E. coli* and *C. glutamicum*, the same procedure was performed with the differential LAPS set-up, as described in Section 3.1 and shown in Figure 2. Figure 4 depicts the corresponding calibration curves with the PCR mean values for variations of the glucose concentration between 0.042 mM and 5 mM for the three studied microorganisms at a constant cell number of 4.8 × 10^9^ cells. For the detailed values, see Table S2 in supplementary information of this article.

In Figure 4, the extracellular acidification of *E. coli* and *C. glutamicum* increases after increasing the glucose concentration, comparable with the results in [27,29]. For *E. coli* and *C. glutamicum* bacteria, the lowest detectable signal change was found for 0.042 mM glucose and for *L. brevis* it was 0.5 mM both at a constant cell number of 4.8 × 10^9^ cells. In experiments with *E. coli* bacteria, the PCR values increased from 0.13 ± 0.07 mV/min to 6.40 ± 0.10 mV/min upon increasing glucose concentrations from 0.042 mM to 5.0 mM. The PCR values of *C. glutamicum* bacteria increased from 0.20 ± 0.08 mV/min to 5.60 ± 0.15 mV/min after raising glucose concentrations, respectively (similar to glucose concentrations for *E. coli* in Figure 4). However, the first detectable PCR value 0.04 ± 0.01 mV/ min for *L. brevis* cells could be obtained at a glucose concentration of 0.50 mM. For lower glucose concentrations (e.g., from 0.042 to 0.4 mM), no detectable signal changes could be monitored. It seems to be that *L. brevis* bacteria require more glucose to be able to form enough H^+^-ions on the LAPS surface (shifted green curve in Figure 4). That is supported by the requirement of a higher amount of glucose (20 g/L) during the cultivation phase compared to *E. coli* (1 g/L) and *C. glutamicum* (5 g/L). The glucose concentrations of the respective culture mediums (see also Section 2.2) are given in protocols of the DSMZ (German Collection of Microorganisms and Cell Culture GmbH). Nevertheless, the highest PCR values of 6.50 ± 0.05 mV/min at 3.33 mM and 7.20 ± 0.06 mV/min at 5.0 mM were found with *L. brevis* bacteria. The plotted calibration curves in Figure 4 follow a kinetic behavior for a capacitive field-effect biosensor, which is exemplary discussed for *E. coli* in [46], similar like the Michaelis–Menten kinetics known for enzymatic reactions. Each calibration curve shows a saturation-like behavior with maximum PCR values (e.g., 7.20 ± 0.06 mV/min for *L. brevis* cells at 5.0 mM glucose), where no higher signal changes were detected when glucose concentration is increased further. In this context, two points might be taken into consideration: 1) after the acidification phase with higher cell numbers, the measured medium on the sensor surface will get more acidic. Due to a change of the physiological conditions in the acidic environment on the sensor surface, the activity of some cells in close proximity to the sensor surface might get blocked. Hence, a detection limit to higher PCR values can be caused; 2) in the measurement chambers (1–3), higher cell numbers in suspensions might inhibit the diffusion of more glucose molecules to the underlying cells on the pH-sensitive transducer layer.

**Figure 5:**
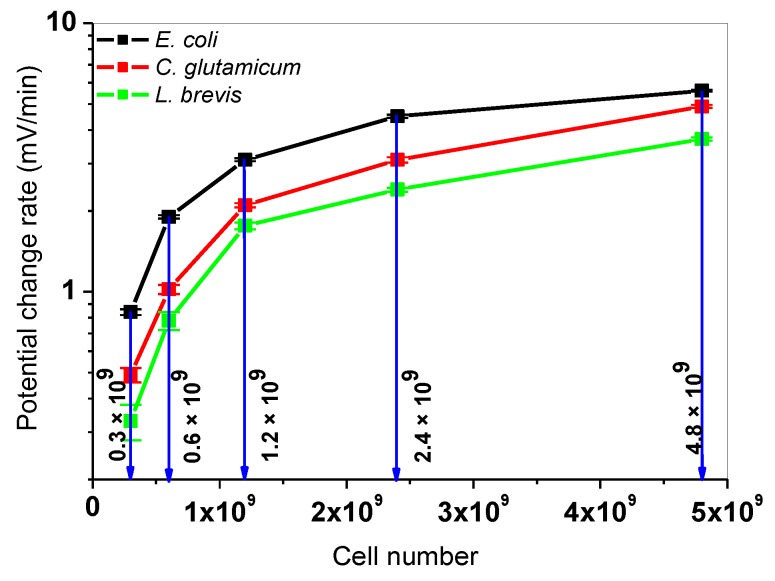
Correlation between the LAPS signal response and the cell number. The mean values and standard deviations of PCR values of *E. coli* (black), *C. glutamicum* (red), and *L. brevis* (green) are depicted for different cell numbers (0.3 × 10^9^, 0.6 × 10^9^, 1.2 × 10^9^, 2.4 × 10^9^, 4.8 × 10^9^ cells) at a constant glucose concentration of 1.67 mM. Three independent measurement repetitions were performed.

In the next step, one glucose concentration (1.67 mM, blue arrow in Figure 4) was selected and all microorganisms were compared in terms of varying cell numbers (0.3 × 10^9^, 0.6 × 10^9^, 1.2 × 10^9^, 2.4 × 10^9^, and 4.8 × 10^9^ cells). Here, five independent successive differential measurements were performed with the four-chamber LAPS set-up. Figure 5 shows the corresponding calibration curves with PCR values of *L. brevis*, *C. glutamicum*, and *E. coli* bacteria.

By increasing the cell number, the total metabolic response of the cells in the suspension increases. As explained for the calibration curves in Figure 4, a saturated-like behavior was observed, too. The highest PCR value of 5.60 ± 0.15 mV/min was obtained with *E. coli* cells at 4.8 × 10^9^ cells. The lowest PCR value of 0.33 ± 0.04 mV/min was calculated for *L. brevis* at 0.3 × 10^9^ cells. For the detailed values, see Table S3 in supplementary information of this article. The results from Section 3.2 show that for three different microorganisms, calibration matrices as function of glucose concentration or cell number can be defined. The obtained results are in good agreement with the results with single cell types on LAPS, which are reported in [27] for *E. coli* K12, in [29] for *C. glutamicum*, and in Section 3.1 for *L. brevis*.

### 3.3. Simultaneous Measurements with L. Brevis, C. Glutamicum, and E. Coli Bacteria

The extracellular acidification of *L. brevis*, *C. glutamicum*, and *E. coli* bacteria was determined simultaneously by means of the differential LAPS set-up. Here, the cell suspensions were parallelly prepared and then loaded into the chambers 1, 2, and 3. The fourth reference chamber was used again without cells. First, the four-chamber sensor arrangement was conditioned with 100 µL of the glucose concentration for 10 min. After this conditioning phase, 200 µL of respective cell numbers were added. In the first chamber *E. coli*, in the second chamber *C. glutamicum*, and in the third chamber *L. brevis* cell suspensions were pipetted. Three measurement repetitions were carried out to verify the reproducibility of all measurements.

**Figure 6:**
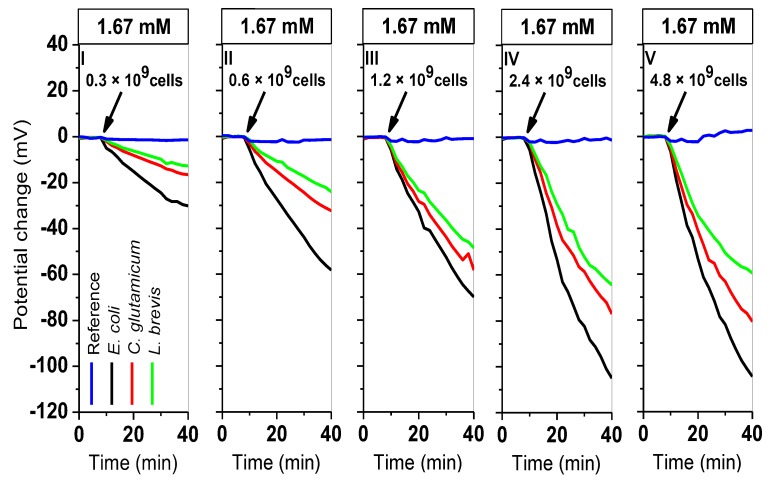
Four-chamber differential LAPS measurement with varying cell numbers of *L. brevis*, *C. glutamicum*, and *E. coli* (0.3 × 10^9^, 0.6 × 10^9^, 1.2 × 10^9^, 2.4 × 10^9^, 4.8 × 10^9^ cells in 200 µL cell suspension) at a constant glucose concentration of 1.67 mM. The potential changes of five successive independent measurements (Nr. I–V) with an increasing number of cells are plotted. Blue line: reference sensor signal without cells; black (*E. coli*), red (*C. glutamicum*), and green (*L. brevis)* lines are active sensor areas with cells. Four laser-diode modules (LDMs) were considered for each chamber (each curve corresponds to the calculated mean values).

For monitoring of the extracellular acidification, five independent, successive measurements (Nr. I–V) were performed (40 min each) by keeping the glucose concentration (1.67 mM) constant and varying the cell number (0.3 × 10^9^, 0.6 × 10^9^, 1.2 × 10^9^, 2.4 × 10^9^, 4.8 × 10^9^ cells). Figure 6 depicts the LAPS signal responses before and after adding cells. After adding cells (10 min after starting each measurement, see diagrams, Nr. I–V), the sensor signal dropped due to an increase of the H^+^-ion activity at the sensor surface.

The signal changes are indicated with different colors (*E. coli* in black, *C. glutamicum* in red, *L. brevis* in green, and the reference signal in blue). In all set of measurements with different cell numbers, *E. coli* bacteria have shown the highest potential changes, followed by *C. glutamicum* and *L. brevis* cells. For instance, in the first set of measurements (I) and at the lowest cell number of 0.3 × 10^9^ cells, a potential change of ≈ 30 mV (ΔpH ≈ 0.6) was observed with *E. coli* cells, following by C*. glutamicum* (≈ 17 mV, ΔpH ≈ 0.32), and *L. brevis* (≈ 13 mV, ΔpH ≈ 0.24); the values were calculated as described in Section 3.1. Correspondingly, in the last set of measurements (V) at the highest cell number of 4.8 × 10^9^ cells, a potential change of ≈ 104 mV (ΔpH ≈ 1.93) was recorded with *E. coli*, ≈ 80 mV (ΔpH ≈ 1.5) with *C. glutamicum*, and ≈ 60 mV (ΔpH ≈ 1.11) for *L. brevis* cells. It was observed that by increasing the cell number, the extracellular acidification of bacteria was increased, as expected in agreement with the measurements where the acidification behavior of the cells was examined separately (see Section 3.1 and Section 3.2). In the presence of higher cell numbers, more acids will be produced, which results in an increase of potential change values.

By the simultaneous evaluation with three different microorganisms on a single LAPS chip a kind of ‘signal pattern’ of the metabolic behavior of cells can be obtained. This way, it is possible to study them under absolute identical boundary conditions, which enables to have comparable results in real time. Furthermore, the measurement time can be reduced, which is advisable for time-critical cell-based measurements. Here, metabolic responses of three microorganisms were successfully evaluated within 40 min, whereas by sequential measuring in Section 3.1, 40 min was required for each model organism.

**Figure 7:**
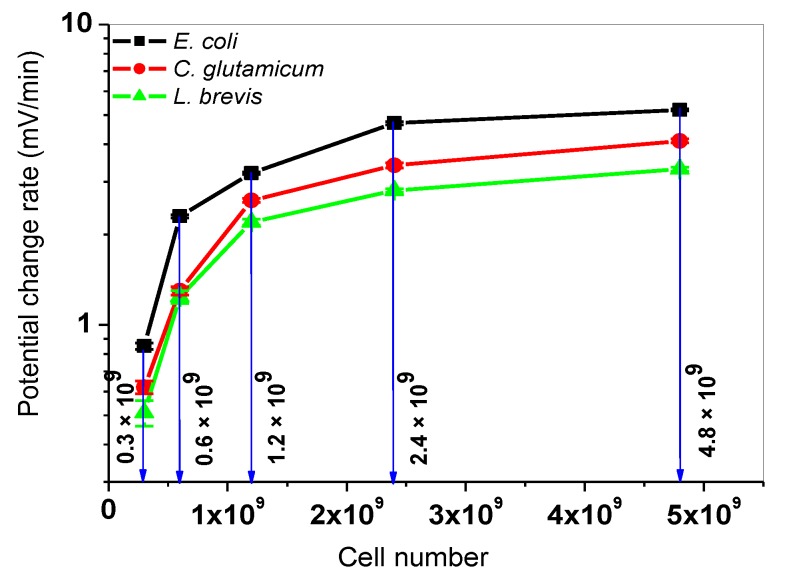
Correlation between the LAPS signal response and the cell number at a fixed glucose concentration of 1.67 mM. Mean values and standard deviations of PCR values of *E. coli* (black), *C. glutamicum* (red), and *L. brevis* (green) are depicted for different cell numbers (0.3 × 10^9^, 0.6 × 10^9^, 1.2 × 10^9^, 2.4 × 10^9^, 4.8 × 10^9^ cells in 200 µL cell suspension). Three independent measurements were performed, and within the first 6 min after adding cells, the mean PCR values were calculated.

The resulting PCR values from Figure 6 were calculated and plotted as calibration curve in Figure 7, which follows a similar behavior, as it was explained for Figure 4 and Figure 5.

The highest PCR value of 5.20 ± 0.04 mV/min was calculated for *E. coli* bacteria for with 4.8 × 10^9^ cells at 1.67 mM glucose. Respectively, the highest PCR values of 4.10 ± 0.06 mV/min (*C. glutamicum*), and 3.30 ± 0.05 mV/min (*L. brevis*) were obtained. The lowest detected PCR values were found at the lowest cell number (0.3 × 10^9^ cells) and 1.67 mM glucose: 0.85 ± 0.02 mV/min (*E. coli*), 0.62 ± 0.03 mV/min (*C. glutamicum*), and 0.51 ± 0.05 mV/min (*L. brevis*). For the detailed values, see Table S4 in supplementary information of this article.

In order to validate whether different microorganisms in simultaneously performed measurements might mutually influence the signal characteristics of each other (cross-talk between four chambers), PCR values between the two experiments (sequential/simultaneous) for *E. coli*, *C. glutamicum*, and *L. brevis* are compared in Table 1; |Sequential PCR|, |Simultaneous PCR| and the difference between the two experiments |Δ PCR| are listed. Starting with a cell number of 0.3 × 10^9^ cells up to 4.8 × 10^9^ cells, signal value differences (|Δ PCR|) were studied: on the one hand, for most cell numbers, independent of the cell type, a good correlation was found with |Δ PCR| ≤ 0.46 mV/min, representing usual variations in preparation of cell suspensions and cultivation process. Such slight deviations in cell numbers will influence the metabolic response and consequently, the extracellular acidification, and finally the sensor output signal. On the other hand, the highest variation was obtained for *C. glutamicum* at 4.8 × 10^9^ cells (0.8 mV/min) and at 1.2 × 10^9^ cells (0.5 mV/min). A possible explanation for this discrepancy might be differences in surviving cell numbers after manual preparation of cell suspensions (in diluted PBS solution) after the cultivation process. Through automatization of the cultivation steps and parallelization of the sample preparation process, PCR value variations between measurements can be further minimized in future experiments. In addition, measurements have also shown that both experimental procedures (sequential/simultaneous measurement) are in good accordance with published data for *E. coli* and *C. glutamicum* bacteria [27,29].

## 4. Conclusions

This article comprises two essential achievements in terms of LAPS-based biosensors: i) for the first time, a cell-based LAPS set-up was utilized to determine the extracellular acidification of *L. brevis* bacteria by varying the two parameters of glucose concentration and cell number. It was observed that by increasing the cell number and/or glucose concentration, the extracellular acidification of *L. brevis* cells increases as expected, due to an increase of H^+^-ion activity on the sensor surface after the acidification phase. The overall sensor characteristic is comparable to data published for *E. coli* and *C. glutamicum*, however, the absolute values of its metabolic response differ, depending on the cell type. ii) For the first time, simultaneous measurements were carried out with three different microorganisms (*E. coli*, *C. glutamicum*, *L. brevis*) on the same LAPS chip. Hence, a ‘signal pattern’ of the extracellular acidification of cells was defined. There was no mutual influence on the signal characteristic for each type of bacteria. Moreover, a good correlation was found between the sequentially and simultaneously performed LAPS investigations.

In future studies, the differential LAPS set-up with model microorganisms can be applied to analyze the metabolic behavior of microorganism populations in (bio)fermentation broths (e.g., in a biogas fermentation broth). With the knowledge about their cellular metabolism obtained by LAPS, the sensor signal patterns with respective calibration curves can be defined. Subsequently, the metabolic ‘interaction’ between the fermentation broth and the particular model microorganisms can be evaluated: It can be examined, whether and how cells in the fermentation broth will influence the extracellular acidification of the analyzed model microorganisms. The related signal variations can be monitored at different process stages of a bioreactor, which might allow a better and faster control in case of process disturbances.

## Figures and Tables

**Table 1:** Comparison of mean values and standard deviations of the potential change rate values obtained from sequential measurements (Figure 5) and simultaneous measurements (Figure 7) after adding cells for *E. coli*, *C. glutamicum*, and *L. brevis*. The glucose concentration of 1.67 mM was kept constant and the cell number was varied from 0.3 × 10^9^ up to 4.8 × 10^9^ cells. |Δ PCR| describes the signal difference between both experiments.

Cells In 200 µL	Type Of Bacteria	|Sequential PCR| (mV/min)	|Simultaneous PCR| (mV/min)	|Δ PCR| (mV/min)
	*E. Coli*	0.84 ± 0.13	0.85 ± 0.02	0.01
0.3 × 10^9^	*C. Glutamicum*	0.49 ± 0.05	0.62 ± 0.03	0.13
	*L. Brevis*	0.33 ± 0.04	0.51 ± 0.05	0.18
	*E. Coli*	1.90 ± 0.12	2.30 ± 0.03	0.40
0.6 × 10^9^	*C. Glutamicum*	1.02 ± 0.04	1.30 ± 0.04	0.28
	*L. Brevis*	0.78 ± 0.06	1.24 ± 0.06	0.46
	*E. Coli*	3.10 ± 0.11	3.20 ± 0.03	0.10
1.2 × 10^9^	*C. Glutamicum*	2.10 ± 0.04	2.60 ± 0.04	0.50
	*L. Brevis*	1.76 ± 0.05	2.20 ± 0.05	0.44
	*E. Coli*	4.50 ± 0.10	4.70 ± 0.06	0.20
2.4 × 10^9^	*C. Glutamicum*	3.10 ± 0.22	3.40 ± 0.07	0.30
	*L. Brevis*	2.40 ± 0.06	2.80 ± 0.04	0.40
	*E. Coli*	5.60 ± 0.15	5.20 ± 0.04	0.40
4.8 × 10^9^	*C. Glutamicum*	4.90 ± 0.04	4.10 ± 0.06	0.80
	*L. Brevis*	3.70 ± 0.02	3.30 ± 0.05	0.40

## Supplementary Materials

The following are available online at https://www.mdpi.com/1424-8220/19/21/4692/s1, Table S1, Table S2, Table S3, Table S4.
