# Supplementary material for: A LAPS-Based Differential Sensor for Parallelized Metabolism Monitoring of Various Bacteria
Source: Sensors (Basel). 2019 Oct 29;19(21):4692. doi: 10.3390/s19214692 (PMC6864667; doi:10.3390/s19214692)
Supplement: Supplementary file 1 [file sensors-19-04692-s001.pdf]

Supplementary Materials

Table 1. Mean values and standard deviations (three repetitions) of the potential change rates obtained from the slope of the differential signals obtained from Figure 2 and calculated within the first 6 min after adding cells. Cell numbers and / or glucose concentrations were varied. See also Figure 3.

Glucose (mM)	Cells In 200 µL	|*L. Brevis* PCR| (mV/min)
-	1.2×10^9	1.76 ± 0.05
1.67	2.4×10^9	2.60 ± 0.06
-	4.8×10^9	3.70 ± 0.02
-	1.2×10^9	2.30 ± 0.02
2.5	2.4×10^9	3.30 ± 0.05
-	4.8×10^9	4.80 ± 0.04
-	1.2×10^9	3.30 ± 0.02
3.33	2.4×10^9	4.90 ± 0.03
-	4.8×10^9	6.50 ± 0.04
-	1.2 × 109	4.70 ± 0.03
5	2.4 × 109	6.00 ± 0.04
-	4.8 × 109	7.20 ± 0.05

Table 2. Mean values and standard deviations (three repetitions) of the potential change rate values obtained from the slope of differential signals calculated within the first 6 min after adding cells. The cell number of 4.8×10^9 cells was kept constant and the glucose concentrations were varied. N.D.S.C.: No Detectable Signal Changes. See also Figure 4.

Glucose (mM)	|*L. Brevis* PCR| (mV/min)	|*C. Glutamicum* PCR| (mV/min)	|*E. Coli* PCR| (mV/min)
0.042	N.D.S.C.	0.20 ± 0.08	0.13 ± 0.07
0.085	N.D.S.C.	0.43 ± 0.06	0.28 ± 0.08
0.17	N.D.S.C.	1.00 ± 0.09	0.60 ± 0.12
0.20	N.D.S.C.	-	-
0.33	N.D.S.C.	2.00 ± 0.27	2.53 ± 0.14
0.40	N.D.S.C.	-	-
0.50	0.04 ± 0.01	3.60 ± 0.40	3.45 ± 0.12
0.68	0.21 ± 0.04	-	-
0.83	0.56 ± 0.07	4.20 ± 0.14	4.28 ± 0.12
1.20	1.60 ± 0.06	-	-
1.67	3.00 ± 0.01	4.90 ± 0.48	5.48 ± 0.08
2.50	4.90 ± 0.05	5.10 ± 0.08	5.82 ± 0.07
3.33	6.50 ± 0.05	5.30 ± 0.13	6.12 ± 0.06
5.0	7.20 ± 0.06	5.60 ± 0.15	6.40 ± 0.10

Table 3. The mean values and standard deviations (three repetitions) of the potential change rates obtained from the slope of differential signals calculated within the first 6 min after adding cells for *L. brevis, C. glutamicum,* and *E. coli*. The glucose concentration of 1.67 mM was kept constant and the cell number was varied from 0.3×10^9 up to 4.8×10^9 cells. See also Figure 5.

Glucose (mM)	Cells In 200 μL	ǀ*L. Brevis* PCRǀ (mV/min)	ǀ*C. Glutamicum* PCRǀ (mV/min)	ǀ*E. Coli* PCRǀ (mV/min)
-	0.3×10^9	0.33 ± 0.04	0.49 ± 0.05	0.84 ± 0.13
-	0.6×10^9	0.78 ± 0.03	1.02 ± 0.10	1.90 ± 0.12
1.67	1.2×10^9	1.76 ± 0.05	2.10 ± 0.11	3.10 ± 0.11
-	2.4×10^9	2.40 ± 0.06	3.10 ± 0.22	4.50 ± 0.10
-	4.8×10^9	3.70 ± 0.02	4.90 ± 0.04	5.60 ± 0.15

Table 4. Mean values and standard deviations (three repetitions) of potential change rate values obtained from the slope of the differential signals calculated within the first 6 min after adding cells for *L. brevis, C. glutamicum,* and *E. coli*. The glucose concentration of 1.67 mM was kept constant and the cell number was varied from 0.3×10^9 up to 4.8×10^9 cells. See also Figure 7.

Glucose (mM)	Cells In 200 μL	ǀ*L. Brevis* PCRǀ (mV/min)	ǀ*C. Glutamicum* PCRǀ (mV/min)	ǀ*E. Coli* PCRǀ (mV/min)
-	0.3×10^9	0.51 ± 0.05	0.62 ± 0.03	0.85 ± 0.02
-	0.6×10^9	1.24 ± 0.06	1.30 ± 0.04	2.30 ± 0.03
1.67	1.2×10^9	2.20 ± 0.05	2.60 ± 0.04	3.20 ± 0.03
-	2.4×10^9	2.80 ± 0.04	3.40 ± 0.07	4.70 ± 0.06
-	4.8×10^9	3.30 ± 0.05	4.10 ± 0.06	5.20 ± 0.04
